# The computational-based structure of Dwarf14 provides evidence for its role as potential strigolactone receptor in plants

**DOI:** 10.1186/1756-0500-5-307

**Published:** 2012-06-19

**Authors:** Noura Gaiji, Francesca Cardinale, Cristina Prandi, Paola Bonfante, Graziella Ranghino

**Affiliations:** 1 Geol sas, Vercelli, Italy; 2 Dipartimento di Colture Arboree, Università di Torino, Torino, Italy; 3 Dipartimento di Chimica, Università di Torino, Torino, Italy; 4 Dipartimento DiBios, Università di Torino, Torino, Italy

## Abstract

**Background:**

Strigolactones (SLs) are recently identified plant hormones modulating root and shoot branching. Besides their endogenous role within the producing organism, SLs are also key molecules in the communication of plants with arbuscular mycorrhizal (AM) fungi and parasitic weeds. In fact SLs are exuded into the rhizosphere where they act as a host-derived signal, stimulating the germination of the seeds of parasitic plants which would not survive in the absence of a host root to colonize. Similarly, their perception by AM fungi causes extensive hyphal branching; this is a prerequisite for effective root colonization, since it increases the number of potential contact points with the host surface. In spite of the crucial and multifaceted biological role of SLs, there is no information on the receptor(s) which bind(s) such active molecules, neither in the producing plants, or in parasitic weeds or AM fungi.

**Results:**

In this work, we applied homology modelling techniques to investigate the structure of the protein encoded by the gene *Dwarf14*, which was first identified in rice as conferring SLs insensitivity when mutated. The best sequence identity was with bacterial RsbQ. Both proteins belong to the superfamily of alpha/beta-fold hydrolases, some members of which play a role in the metabolism or signalling of plant hormones. The Dwarf14 (D14) structure was refined by means of molecular dynamics simulations. In order to support the hypothesis that D14 could be an endogenous SLs receptor, we performed docking experiments with a natural ligand.

**Conclusions:**

It is suggested that D14 interacts with and thereby may act as a receptor for SLs in plants. This hypothesis offers a starting point to experimentally study the mechanism of its activity *in vivo* by means of structural, molecular and genetic approaches. Lastly, knowledge of the putative receptor structure will boost the research on analogues of the natural substrates as required for agricultural applications.

## Background

Strigolactones (SLs) are a group of plant-produced carotenoid-derived terpenoid lactones that have been recently implicated in the regulation of shoot and root branching [1-3]. Already long before then, SLs were known as root-exuded molecules capable to provoke the germination of seeds from parasitic plants, like *Striga* and *Orobanche*[4] and, more recently, their role was extended to the induction of hyphal branching and of a burst of mitochondrial activity in arbuscular mycorrhizal fungi (AMF) [5,6]. In undisturbed ecosystems, most plants are colonized by AMF [7], a group of soil-borne fungal endophytes belonging to the ancient *Glomeromycota* phylum. The association is the result of co-evolution events dating back to the early Devonian times [8]: its success in time and space is mostly due to the nutritional benefits both partners gain. How plants and AMF establish a molecular dialogue which eventually allows the symbiosis, is a crucial question in plant biology. The release of soluble signals in the rhizosphere was suggested as an easy solution for both partners to be timely informed of the presence of each other, even before physical contact [9]. AMF produce several bioactive compounds with a chitin-based structure called “Myc factors” [10,11]. At the same time they respond with profuse branching to root exudates from compatible hosts [12,13], whose bioactive molecules are SLs [14]. The ability to perceive SLs has been very recently expanded also to non-AMF, phytopathogenic filamentous fungi [15]. The ability to synthesize SLs is widespread along the plant taxa, also including non-host plants for AMF like Arabidopsis [2].

The characterization of these versatile molecules, the identification of their biosynthetic genes in plants and of their receptors in plants and fungi is at the moment a hot spot in plant biology [16-18]. In spite of the increasing knowledge on SLs synthesis and mechanism of action, the proteins that mediate their perception within the plant are still poorly known. Only two genes, encoding an F-Box protein and a predicted α/β-fold hydrolase, have been identified so far as potentially involved in the perception and/or transduction mechanisms of the SLs signal, since their mutants are SL-insensitive [16,19]. As far as the proteins that are expected to perceive SLs in parasitic plants, even less is known [17]. These studies open the question whether similar proteins with analogous functions may be present in the AMF, but on this respect, no genetic or biochemical information is available as yet. Nevertheless, Akiyama and co-workers [20] have highlighted the molecular structural requirements that specifically correlate with the activity of SLs in stimulating AMF branching *vs.* seed germination in parasitic plants. The incomplete overlap between such requirements makes it likely that the nature of SLs receptors is different in organisms belonging to different kingdoms, whereas it can be envisaged that SLs receptors in parasitic plants may share similarity with the receptor(s) for endogenously produced SLs.

To identify the structure of a potential SLs receptor in plants we used multiple bioinformatics and computational approaches. These allowed us to propose a model compatible with the protein described in rice as D14, and to verify whether its docking features fit to a natural reference SLs molecule (Strigol). A similar approach was applied to GID1 [21] and SABP2 [22], two plant proteins belonging to the same super-family as D14 and broadly involved in the metabolism and perception of two important classes of plant hormones, i.e. gibberellins and salicylic acid with its derivatives.

## Results and discussion

### Sequence analysis and homology modelling of D14

**Figure 1:**
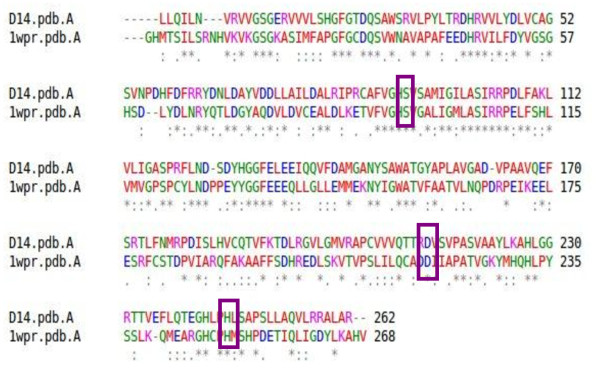
Alignment of D14 and 1WOM (RsbQ). Residues Ser95, Asp219, His250, forming the catalytic triad of RsbQ are conserved in D14.

The *Dwarf14* gene product, D14, is a 318 amino acid protein whose activity and structure are not known yet. However, the phenotype conferred by its known mutant allele in rice resembles the phenotype of SL-deficient mutants. Since this phenotype cannot be rescued by exogenous SLs, the function of D14 is compatible with a role in SLs perception rather than in their synthesis. As a first step we performed multiple-sequence alignment of the D14 sequence with BLAST [23] and ClustalW [24]. Among all the sequences deposited in PDB [25], the one showing the best score was RsbQ, with 38% of identity with the D14 sequence. The alignment of the two sequences is displayed in Figure 1.

RsbQ belongs to the α/β-fold hydrolase super-family and is involved in the stress response of *Bacillus subtilis*[26,27]. The 3D structure of the RsbQ protein was solved by X-ray crystallography and two 3D structures are reported in PDB: the native [PDB: 1WOM] and the inhibitor-bound one [PDB: 1WPR], the inhibitor being phenylmethanesulfonic acid (PMSF). Both were used as templates for the generation of the 3D model of D14 with three different methods, in order to prevent methodological biases (see Methods). The catalytic nucleophile, Ser96, has the same position in both the native and the PMSF-bound structure. This position of the nucleophile is shared among the α/β hydrolases and is due to the formation of a sharp turn, called the “nucleophile elbow” [28,29]. The catalytic triad is buried inside the molecule and the active site is a hydrophobic cavity that is nearly isolated from the solvent. It is inferred from this feature that the catalytic site of RsbQ has specificity for a hydrophobic, small compound, rather than a macromolecule such as RsbP (a protein phosphatase physically interacting with RsbQ and involved in its signalling pathway). Instead, structural comparison with other α/β hydrolases demonstrates that a unique loop region of RsbQ is a likely candidate for the interaction site with RsbP, and that this interaction might be responsible for the product release by operating the hydrophobic gate between the cavity and the solvent.

### Structural refinement and stability evaluation by Molecular Dynamics of the D14 model

Having built the 3D structure of D14 by homology modelling, we noticed that the catalytic triad of RsbQ (Ser95, Asp219, His250) almost overlaps in the active site of the modelled D14. The homologous structure is then optimized without restraints by means of molecular mechanics (MM), in explicit solvent.

**Figure 2:**
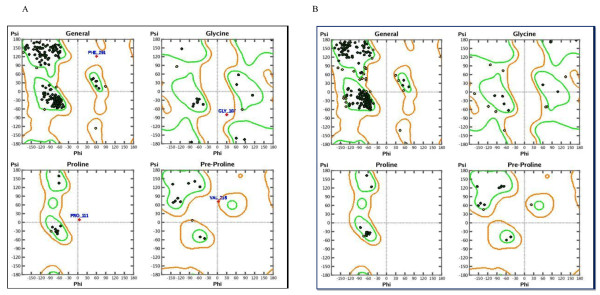
Ramachandran plot of D14 before and after the molecular mechanics.

The geometry of the final refined model evaluated with Ramachandran’s plot calculations computed with the MOE program [29], shows that almost all residues are located within the most favourable, additionally allowed, and generously allowed regions of the Ramachandran’s plot (Figure 2). We then relaxed the D14 structure modelled on 1WPR, without restraining the atomic positions, by means of molecular dynamics (MD) using GROMACS 3.3.3 [30]. After equilibrating the system, a 5 ns (ns) production simulation is conducted with a 1 femtosecond (fs) time step at a pressure of 1 bar and a temperature of 300 K.

**Figure 3:**
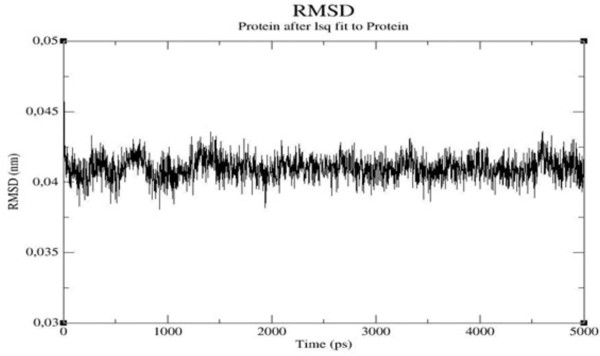
Root mean square deviation between the backbones of the Homolog D14 and the template protein RsBQ during the simulation.

**Figure 4:**
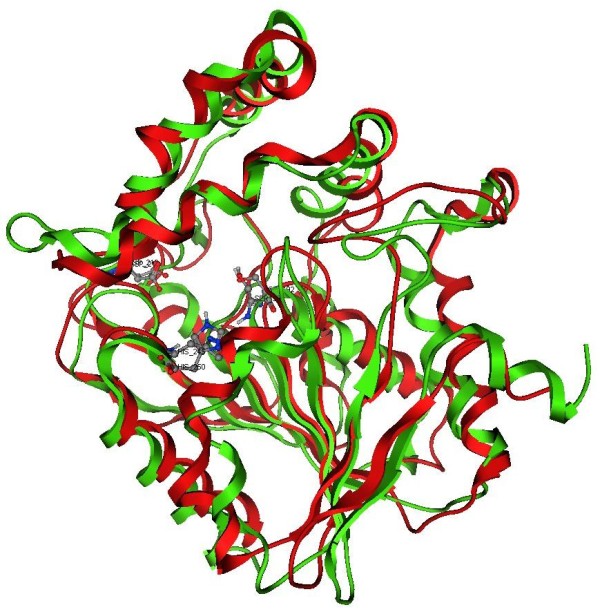
**Superposition of the model of D14: in red and the structure of native RsbQ (1WOM) in green.** The side chains of the catalytic triad are displayed in ball and sticks.

The stability of the macromolecule is checked by monitoring the total energy of the system and the Root Mean Square Deviation (RMSD, Figure 3) of the C-α atoms as a function of time: the global structure of the protein remains quite similar to the initial one and the energy and RMSD (.4 Å) profiles confirm its stability. The protein structure averaged over the last nanosecond of simulation of MD is shown in Figure 4 superposed to the native RsbQ structure [PDB: 1WOM].

The binding site of D14 is elucidated by means of the MOE site-finder module [29] and by using the structure of RsbQ bound to the inhibitor PMSF [PDB: 1WPR] as target.

**Figure 5:**
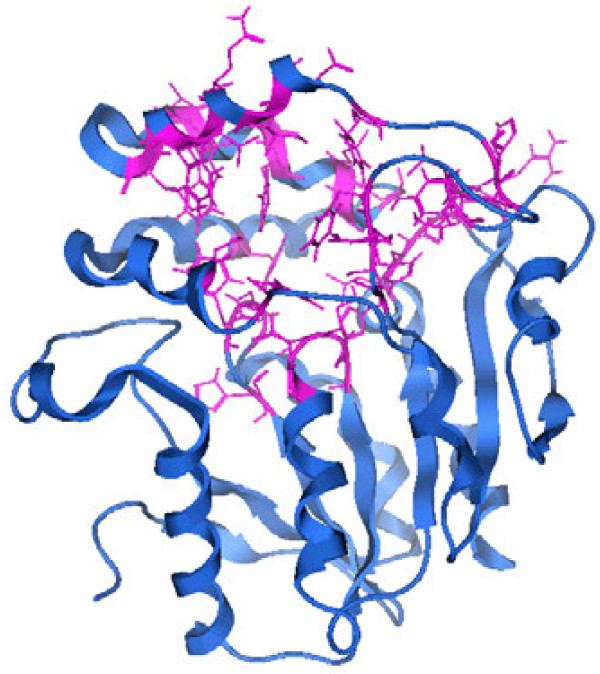
The binding site of D14. In pink the backbone of the residues composing the binding site.

In order to get insights in the configuration and flexibility of the binding pocket during the MD simulation, we performed a second MD simulation of D14 with the SL molecule Strigol docked in the binding site, following the same methodological protocol as above. During both simulations (modelled D14 with and without Strigol) the analysis of the essential dynamics indicated the presence of only one cluster, meaning that there is no conformational diversity and a single representative structure can be used for docking. The final RMSD between the whole model structure [PDB: 1WPR] and the template is 2.17 Å. The binding site has a volume of 223 Å^3^ and the residues that are involved not considering the three catalytic residues, are: His21, Phe23, Gly24, Thr25, His91, Ser92, Ser94, Ser118, Arg120, Phe121, Tyr127, His128, Phe131, Glu135, Ile136, Gln138, Val139, Phe140, Ala142, Met143, Ala149, Trp150, Gly153, Tyr154, Leu157, Ala158, Gly160, Phe170, Cys186, Phe190, Arg212, Asp213, Val214, Ser215, Glu240, His242, Leu243: Figure 5 shows the side chains in purple.

The docking, both flexible and rigid, of Strigol into the site of the protein structure averaged over the last 2 ns of MD of the complex D14-solvent was performed. The putative ligand is well arranged in the site, as already evidenced by MD, and the binding energy is very favourable (−11.3 Kcal/mol). This supports the idea that, similarly to what suggested by the authors in the case of RsbQ, the cognate ligand of D14 could be a small hydrophobic molecule such as SLs.

### D14 and the Hydrolases mechanism

**Figure 6:**
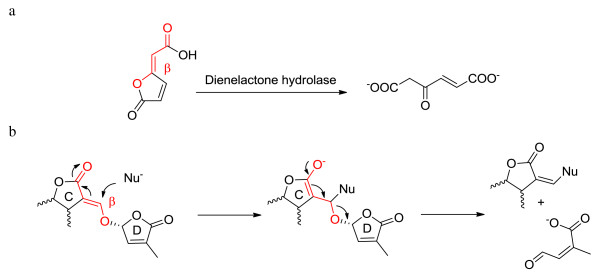
a: The reaction catalyzed by Dienelactone Hydrolase; b: Mechanism for SLs induction of seed germination occurring at the receptor site.

RsbQ [PDB: 1WPR] has the very well defined α/β fold of hydrolases. The proteins having such a fold form a super-family of structurally related enzymes with diverse catalytic functions and in some cases, no enzymatic activity. The enzymes all have a Nucleophile-His-Acid catalytic triad that operates on substrates with different chemical composition and in various biological contexts. Mutations involving the catalytic triad influence activity, but they do not influence the overall 3D structure. Members in this super-family include, among many others, dienelactone hydrolases (DLHs) [31,32]. The reaction catalyzed by DLHs is represented in Figure 6.

**Figure 7:**
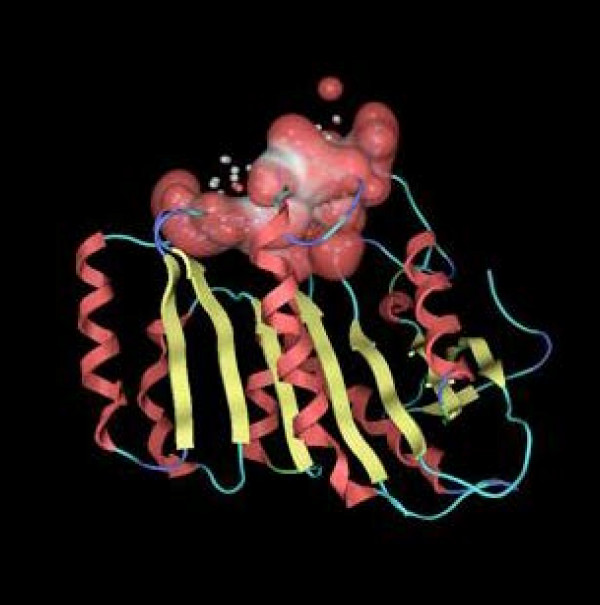
Active site of DLH is displayed with red-white Van der Waals accessible surface.

**Figure 8:**
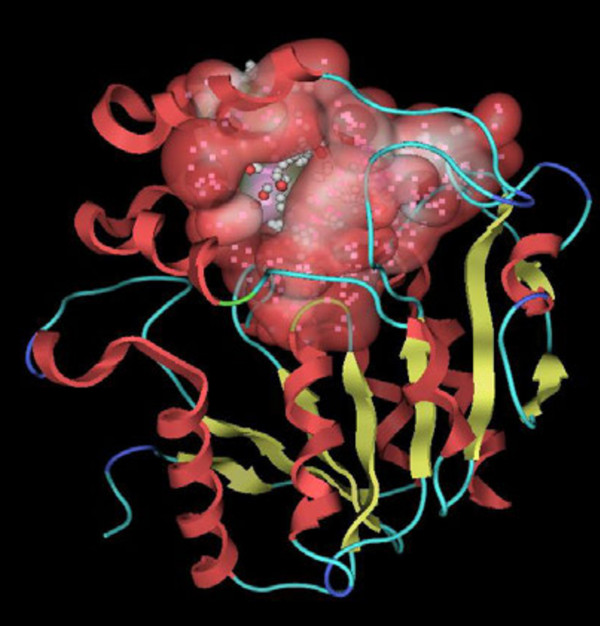
Active site of D14 is displayed with red-white accessible surface.

According to the literature [33], DLHs promote the conversion of the dienelactone to an open chain product. Interestingly, the bioactiphore of both natural and synthetic SLs - that has been demonstrated to be the enol ether bridge connecting the C and D rings conjugated with the carbonyl of the C ring (highlighted in red in Figure 6) - shows similarities with the substrate of DLHs. A possible general mechanism at the base of the bioactivity of SLs was proposed by Zwanenburg [17]. In this case a nucleophilic site present in the receptor pocket attacks the β position of the enol ether inducing an addition-elimination process eventually leading to the opening of the butenolide D-ring. If the natural D14 substrates have the same functional groups as SLs, then the D14 protein could actually be part of the family of DLHs. The structure of a DLH from *Pseudomonas* sp. B13 is present in the PDB database [PDB: 1DIN] and is defined as a α/β-hydrolase enzyme with lactonic type of substrate [32]. Other available structures are obtained by site-directed mutagenesis of the same 1DIN. DLHs contain seven α helices and eight strands of β-pleated sheets. Cys123, His202 and Asp171 form the catalytic triad. One of the active mutant structures is Cys123Ser, as in the catalytic triad of D14. A single 4-turn 3(10)-helix is present (see Figure 1 of reference [32] for the common hydrolases topology). The active-site Cys123 resides at the N-terminal end of the α helix that is peculiar as it consists entirely of hydrophobic residues. A conformational change is postulated when the ligand binds to the active site of the enzyme (visible as Connolly’s dot surface in Figure 7). The structures of a DLH and of D14 are shown in Figures 7 and 8; their comparison highlights a very similar fold and a conserved active site, the main difference being in the cap that closes the access to the active site in D14. The DLH has a short α helix that could play the same role of hinge bending on the active site as mentioned above, but this is anyway much shorter than the one in D14.

Having postulated the same enzymatic activity for D14 and DLHs, we focused on the binding mode and energy of both the putative reactant and product. We performed a docking of Strigol on D14 and the DLH mutant Cys123Ser, hypothesizing a DLH-like reaction, as -outlined in Figure 6, occurring in the active site. Docking energy values of substrate and product for DLH-like reaction are in Table 1

**Table 1:** Docking energies of substrate and product for the DLH-like reaction

	**D14**	**DLH Cys123Ser**
Substrate (Strigol)	−8,20 kcal/mol	−8,71 kcal/mol
Product	−12,70 kcal/mol	−10,42 kcal/mol

Both structural and catalytic features are consistently in agreement with the hypothesis put forward for D14, that is, D14 can bind to SLs and modify their structure by opening the lactone ring.

### Comparison between D14 and other proteins playing a role in plant defence, development and metabolism

**Figure 9:**
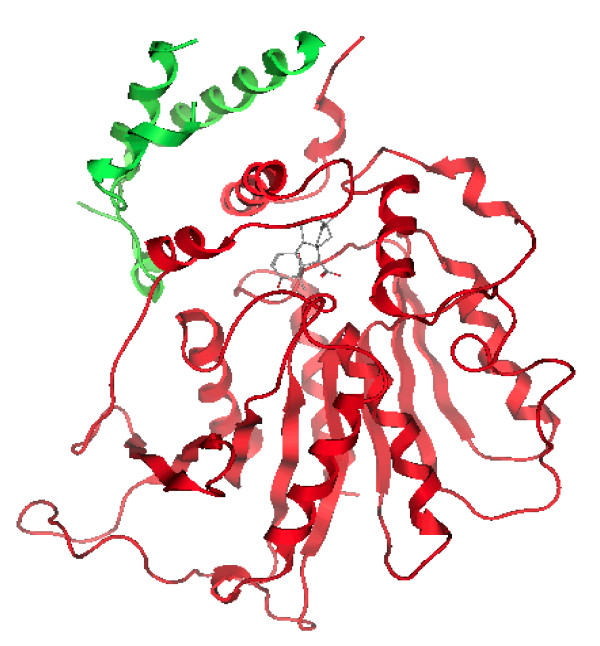
**3D structure of the gibberellin receptor: GID1A (Red) in complex with DELLA (green) protein, and gibberellin in the binding site.** DELLA proteins are functional partners of GID1A and participate in the signal transduction process downstream of gibberellin perception.

**Figure 10:**
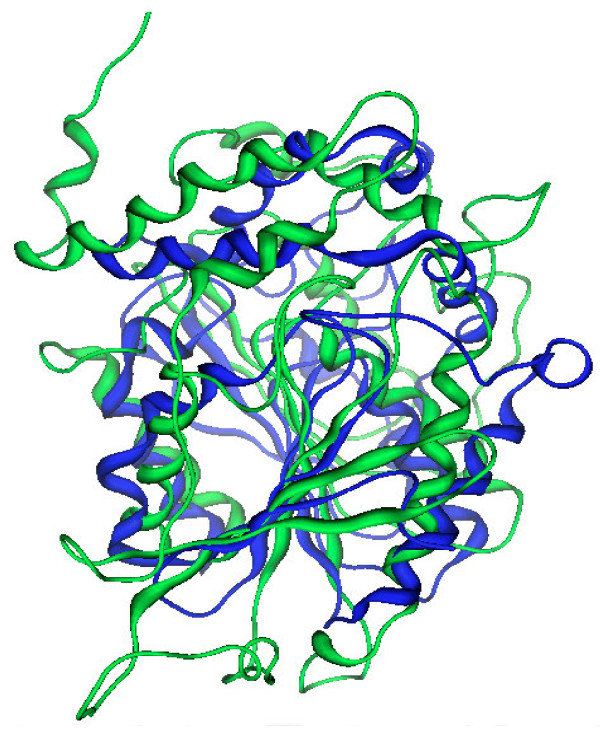
Superposition of GID1A (2ZSH, green) and D14 (blue).

Gibberellins are plant hormones involved in the shaping of plant architecture and in the reaction to environmental cues, microbial pathogens included [34,35]. The structure of GID1, the ligand-interacting subunit of the gibberellin receptor complex, was obtained by X-ray diffraction [PDB: 2ZSH]. It shows a two-chains assembly: the so called chain A shows a α/β-hydrolase fold and contains the binding site for gibberellin A (Figure 9). D14 and GID1A share only 8% of sequence identity. The catalytic triad of GID1A, for which no enzymatic activity has been reported, is Ser116, Asp289 and Val319. The active site arrangement and dimensions are larger than in D14. Superposition of GID1A (green) and D14 (blue) has an RMSD 10.70 Ǻ (Figure 10). Altogether the two structures have little in common, albeit sharing a similar fold. However the paper reporting the structure of GID1A points out that this receptor component shares a good sequence similarity with two esterases [21].

We hence compared D14 and the enzyme showing the highest sequence similarity to GID1A: this is tobacco SABP2 [PDB: 1Y7H], a methylsalicylate (MeSA) esterase. The product, salicylic acid (SA), is a critical signal for the activation of plant defence responses against pathogen infections. SABP2 is thought to convert MeSA to SA as part of the signal transduction pathways that activate systemic acquired resistance and perhaps local defence responses as well [35].

**Figure 11:**
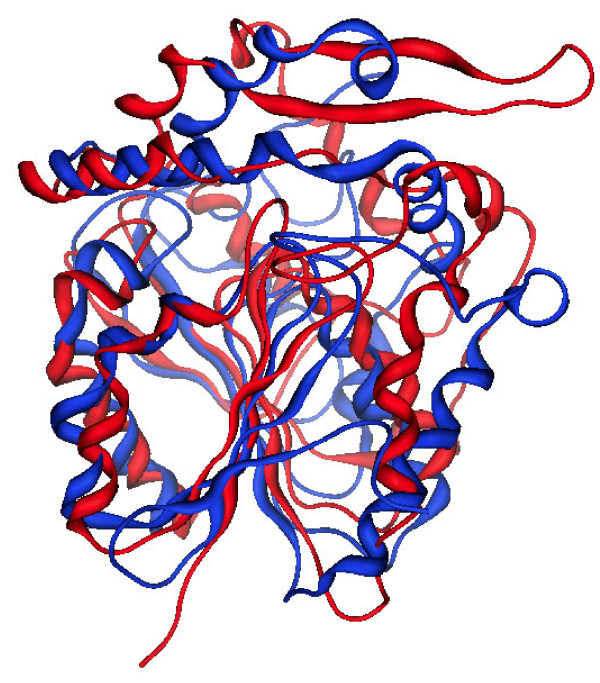
Superposition of SABP2 (1Y7H, red) and D14 (blue).

Despite the fact that D14 and SABP2 share only 18% of sequence identity, they have very similar 3D folds and binding sites (Figure 11). Superposition of SABP2 (red) and D14 (blue) has a RMSD of 5.46 Å. SABP2 itself is in fact a member of the α/β-hydrolase super-family of enzymes, with Ser81, His238, and Asp210 as the catalytic triad. SABP2 has strong esterase activity with MeSA as the substrate, and the product SA is a potent product inhibitor of this catalysis, being bound in the active site and completely shielded from the solvent. The product of a DLH reaction by D14 on SLs could also be locked into the active site, as indicated by the docking energy of the product that is more favourable than that of the substrate (see above Table 1).

The structural comparison between SABP2, DLH and D14 supports an analogy between their enzymatic activity as esterases: the figures evidence the good fit among the α/β fold and the diversity in the αF-chain and loop that may in fact recognize the substrates or act as lids in the signal transduction pathway.

## Conclusions

The aim of this work was to describe the structure of the protein D14, which is encoded by the gene *Dwarf14* and was suggested to be a potential SLs receptor in plants [16]. From the structural comparison of D14 with a bacterial protein sharing 38% of sequence identity (RsbQ), it is inferred that SL signalling may involve a step with a hydrolasic-like catalytic mechanism: this is consistent with the structural requirements for SLs molecules active in parasitic plants.

Indeed, the detailed comparison of D14 with known structures shows that the active site is highly similar to that of DLHs. However in the case of D14, the pocket is not exposed to solvent, but protected by a helical domain that appears to be flexible: its hinge movement could be related to the recognition of the substrate and be part of the signal transmission by means of a conformational movement. This mechanism has been already postulated for the recognition of gibberellins by GID1 [21]: however the binding sites of D14 and GID1 are different, and both their sequences and 3D structures have a low degree of similarity. Instead, high similarity is found between D14 and a known plant esterase, SABP2. This is involved in the processing of MeSA to obtain SA, a molecule controlling a subset of stress responses in plants. Once again, 3D structures of D14 and SABP2 are well correlated, and the demonstrated (SABP2) and postulated (D14, this work) enzymatic activities show intriguing analogies.

In conclusion, our results on one hand confirm previous findings suggesting that D14 can be regarded as a receptor for SLs in plants. On the other hand, they provide the first computational reconstruction of the 3D structure of D14 offering a model to be tested for experimental studies of *in vitro* and *in vivo* activity by means of structural, molecular and genetic approaches. The knowledge of the putative receptor structure will boost the research on analogues of the natural substrates as required for agricultural applications.

## Methods

### Homology modelling

The aminoacidic sequence of Dwarf14 (D14) was taken from the article of Arite et al. [16]. Multiple sequences alignment was performed using the ClustalW program accessible on-line through the European Bioinformatics Institute [24] and BLAST [23]. A model of the D14 protein was generated using SWISS MODEL [36] and EsyPred3D [37]. The model structure was based on the files PBD: 1WP0 and 1WPR; these template proteins, belonging to the family of α/β hydrolases, were chosen because of a significant sequence similarity with D14, in addition to their satisfactory crystallographic resolution. The model was subsequently verified using MOE-ProEval, an implementation of the PROCHECK suite of stereochemical measurements, and Ramachandran’s maps [29].

### Molecular Dynamics simulations

The 3-D molecule was locally minimized *in vacuo* by constraining the backbone to the template molecule in order to give a first optimization of the rough geometry derived from homology modeling, particularly for the side chains and the added polar hydrogen atoms.

GROMACS [30,38] was then used for MD simulations. The structure of D14 was inserted into a cubic box maintaining a minimum of 9 Å between the box edges and the protein surface. The resulting system was solvated with Simple Point Charge (SPC) water molecules provided in the GROMACS package and then minimized with the GROMOS96 force field using the steepest descent method in order to lead the system to a more favourable energetic condition before starting the MD simulation. The temperature of the bath was set to 300 K and the coupling time constant was set to 0.1 ps. The box pressure was maintained at 1 bar using 1ps time constant and a water compressibility of 4.5 × 10–^5^ bar^−1^. Coulombic interactions were treated with the PME (Particle Mesh Ewald algorithm) model with a cutoff of 1.6 nm. Configurations were saved every 100 fs for analysis. After equilibrating the system, a 5 ns production simulation was conducted with a 1 fs time-step at a pressure of 1 bar and a temperature of 300 K. At this stage no constraints or restraints to the template structure 1WOM were added. The only constraint applied was to the α-helices and β -sheets H bonds, using the LINCS [39] algorithm: this is an algorithm that resets bonds to their correct lengths after an unconstrained update. The following parameters were used: lincs-order of 4, lincs-warn angle of 30 and unconstrained start. Computer simulations describe protein dynamics, and under the limit of their accuracy and extension, they should contain information on functional motion and ability to address the relationship that motion has with structure. Essential dynamics (ED) [40] has been a fairly applied method to extract useful information from protein simulation. In particular the ED analysis reveals high-amplitude concerted motions in the equilibrated portion of the trajectories, based on the diagonalization of the Cα covariance matrix of the atomic positional fluctuations. The collection of the selected eigenvectors describing the collective motions is termed “essential subspace” and can describe protein motions at a reasonable level of accuracy. Correlation plots were obtained by first computing Cα correlation matrices [41]*C(i,j)*, where C*(i,j)* is the covariance matrix of protein fluctuations between residues *i* and *j*.

### Binding-site identification and analysis

The Site Finder module of MOE 2008.10 [29] was used to identify the putative binding pockets and protein ligand-binding sites in the energy-minimized 3D structure of D14. The Site Finder module of MOE 2008.10 generates hydrophobic and hydrophilic alpha spheres serving as probes denoting zones of tight atom packing. These alpha spheres are then used as centroids for the creation of dummy atoms used to define potential binding sites [29].

### Ligand Docking

The average structure of D14 resulting from the last 2 ns of molecular dynamics with the Strigol molecule in the binding pocket was used to carry out ligand-receptor simulations. The molecular docking simulations were performed with MOE dock package [29] and Delos package (N. Gaiji, F. Archetti, P.C. Fantucci, E.L. Zimolo, L. Roggia DELOS: Method of construction and selection of virtual libraries in combinatorial chemistry. European Patent Application EP1628234, holder: Università Milano Bicocca). The ligand explores the conformational space to locate the most favourable binding orientation and conformation by aligning and matching all triangles of the template points with compatible geometry, while the protein atoms remain fixed. An affinity scoring function, ΔG, was employed to rank candidate poses. This simulation is divided into three stages: 1. Conformational analysis, during which ligand is treated in a flexible manner by rotating rotatable bonds. 2. Placement, during which a collection of orientations is generated from the pool of ligand conformations. In this case, the alpha-triangle placement method was used, which generates orientations by superposition of ligand atom triplets and triplet points in the receptor site. The receptor site points are alpha sphere centres which represent locations of tight packing. At each iteration, a random conformation is selected; a random triplet of ligand atoms and a random triplet of alpha sphere centres are used to determine the orientation. 3. Scoring, during which each orientation generated by the placement methodology is subjected to scoring in an effort to identify the most favourable orientations.

## Abbreviations

AMF, Arbuscular mycorrhizal fungi; D14, Dwarf14; DLH, Dienelactone hydrolase; ED, Essential dynamics; GID1, Gibberellin insensitive 1; MeSA, Methyl salicylate; MD, Molecular dynamics; MM, Molecular mechanics; PME, Particle mesh ewald algorithm; PMSF, Phenylmethane sulfonic acid; RMSD, Root mean square deviation; RsbP, Regulator of sigma (b) P; RsbQ, Regulator of sigma (b) Q; SA, Salicylic acid; SABP2, Salicylic acid-binding protein 2; SLs, Strigolactones; SPC, Simple point charge.

## Competing interests

The authors declare that they have no competing interests.

## Authors’ contributions

NG carried out homology-modelling and molecular-dynamics experiments, binding-site and ligand-docking studies; and drafted the manuscript. FC and CP participated in the design of the study and in the interpretation of results, and helped to revise the manuscript. PB was involved in the experimental design. GR conceived of the study, participated in its design, execution and coordination, and helped to draft the manuscript. All authors read and approved the final manuscript.

## Authors’ information

NG and GR are researcher and head of scientific projects, respectively, at Geol Sas – via Monte Bo, 2 – 13100 Vercelli

FC is staff researcher in Plant Physiology at the Dept. of Arboriculture, Università di Torino – via L. da Vinci, 44 – 10095 Grugliasco (TO) Italy

CP is associate professor of Organic Chemistry at the Dept. of Chemistry, Università di Torino – via P. Giuria, 7 – 10125 Turin

PB is full professor of Plant Biology at the Dept. of Life Science and Systems Biology, Università di Torino – viale P. Mattioli, 25–10025 Turin, Italy.
